# Iron and folic acid supplementation compliance during pregnancy and its effect on post-pregnancy anaemia among reproductive-age women in East Africa

**DOI:** 10.1177/17455057251317547

**Published:** 2025-02-10

**Authors:** Melaku Tadege Engidaw, Patricia Lee, Faruk Ahmed

**Affiliations:** 1 Public Health, School of Medicine and Dentistry, Gold Coast Campus, Griffith University, Gold Coast, QLD, Australia; 2 Department of Public Health (Human Nutrition), College of Health Sciences, Debre Tabor University, Debre Tabor, Ethiopia; 3 Department of Medical Research, China Medical University Hospital, Taichung City, Taiwan

**Keywords:** compliance, IFA supplementation, anaemia, women, factors, East Africa

## Abstract

**Background:**

Despite the government’s effort to reduce the prevalence of anaemia among reproductive-age women globally, it continues as a significant public health issue, especially in low- and middle-income countries. Iron–folic acid (IFA) supplementation is a widely used intervention to prevent anaemia, but compliance remains a major challenge. There is a lack of literature examining IFA supplementation compliance during pregnancy and its impact on preventing and controlling anaemia during post-pregnancy in the East Africa region.

**Objective:**

This study aims to investigate compliance with IFA supplementation during pregnancy and its impact on post-pregnancy anaemia among reproductive-age women in East Africa.

**Design:**

This study was designed as a cross-sectional survey.

**Methods:**

This study used data from 43,200 reproductive-age women from 2015 to 2022 demographic and health survey datasets. We used multilevel mixed-effect logistic regression analysis to identify associated factors with IFA supplementation compliance during pregnancy. Furthermore, a propensity score matching (PSM) analysis was used to determine the effect of IFA supplementation compliance on anaemia after pregnancy among women.

**Results:**

The level of compliance with IFA supplementation during pregnancy was 31.33% (95% confidence interval (CI): 30.89, 31.77), whereas the prevalence of anaemia among reproductive-age women was 32.08% (95% CI: 31.64, 32.52). Maternal education, the timing of antenatal care (ANC) visits, wanted index pregnancy (last pregnancy), wealth status, healthcare access, mass media exposure and ANC services from skilled healthcare providers were significantly associated with compliance with IFA supplementation among pregnant women. The PSM analysis indicated a significant positive association between IFA supplementation compliance during pregnancy and prevention of post-pregnancy anaemia among reproductive-age women, with an average treatment effect on the treated (ATT) of 25.55% (ATT = −0.2555, 95% CI: −0.3440, −0.1669, *p* < 0.0001).

**Conclusion:**

Despite universal IFA supplementation, anaemia remains prevalent in East African countries due to low level of compliance with IFA supplementation. Targeting individual and socio-economic factors during IFA supplementation promotion helps to prevent anaemia after pregnancy. Further research is recommended to gain deeper insights.

## Introduction

In 2019, almost 30% of women aged 15–49 across the globe experienced anaemia, with a prevalence of 29.6% in non-pregnant women and 36.5% in pregnant women.^1^ The problem is more prominent in developing countries like East African countries, where the most vulnerable populations are located.^1,2^ While the aetiology of anaemia is often multifactorial, it is commonly caused by inadequate iron-rich food intake,^3^ high rates of infections^4–6^ and inadequate intake of vitamins.^7^ Other causes include genetic defects (like Thalassemia and sickle cell anaemia), certain medicines and immunologic and non-infectious diseases (cancer).^8–10^

In sub-Saharan Africa, anaemia among reproductive-age women is primarily caused by iron deficiency.^11^ Anaemia during pregnancy leads to inadequate weight gain during pregnancy, preterm labour, placenta problems, cardiac complications, haemorrhage, high risk of infection and reduced physical work capacity. It also poses risks to newborns like premature birth, low birth weight, intrauterine growth restriction and childhood anaemia.^12–15^ In addition, folic acid deficiency during conception and early pregnancy is linked to foetal malformations (like neural tube defects), preeclampsia and preterm delivery.^16,17^

Most low- and middle-income countries (LMICs) have implemented the universal iron–folic acid (IFA) supplementation programme as a critical intervention to prevent and control anaemia before and during pregnancy.^18–21^ As a result, free IFA supplementation is included in free maternal nutrition services and supplies in public healthcare systems for all pregnant women to prevent anaemia and reduce adverse pregnancy outcomes in those countries.^22,23^

To address this, the World Health Organization (WHO) recommends that all pregnant women, in settings where anaemia is a public health problem, take 30–60 mg of elemental iron and 400 μg of folic acid orally once daily.^19^ Furthermore, the WHO recommends taking 90 or more IFA supplementation tablets/capsules during the pregnancy period.^19,24–26^ However, IFA supplement intake during pregnancy is low in LMICs,^27,28^ with a study in African countries revealing that only 12% of pregnant women^29^ and none of those planning to be pregnant^30^ consumed the recommended IFA supplementation dosage.

Furthermore, in Africa, due to a lack of advocacy work on anaemia prevention, benefits of IFA supplementation and adequate intake of iron-rich foods, the prevalence of anaemia during and after pregnancy continues as a public health problem.^31^ Currently, most developing countries have adopted a universal IFA supplementation programme for pregnant women through health facilities,^18^ but this is affected by the late initiation of antenatal care (ANC) visits, difficulty in access and insufficient supplies in health facilities.^32–34^ Consequently, most women arrive at health institutions to receive ANC services after developing anaemia and lack adequate time to take the recommended number of IFA tablets.

Despite the availability of free IFA supplementation in many developing countries including East Africa, compliance with the recommended dosages remains remarkably low, making it challenging to effectively reduce maternal anaemia.^27,28,35,36^ The main reasons for the low compliance with IFA supplementation tablets include forgetfulness, temporary side effects caused by the tablets, misconceptions, insufficient knowledge, socio-demographic factors, infrequent use of ANC, inadequate counselling during follow-up, limited healthcare provider awareness, long distance from home to health institution and poor communication about the availability of the services.^37–42^

Besides these challenges, the goal of IFA supplementation is to prevent and control anaemia during pregnancy, build iron reserves for the postpartum period and ensure both the mother and child meet their iron needs after birth.^19,28^ In developing countries, 50%–80% of postnatal women are anaemic due to poor compliance and inadequate micronutrient intake.^43^ Replenishing iron lost during pregnancy and childbirth is crucial, as untreated postnatal anaemia can have severe consequences for both mother and infant.^44–46^ Postpartum anaemia remains a significant public health concern that can be prevented and treated with IFA supplementation.^47^

As mentioned earlier, improving IFA supplementation compliance is essential for adequate micronutrient intake, preventing anaemia and building iron reserves during and after pregnancy, which may help break the intergenerational cycle of nutrition-related health problems.^18,26,48^ Despite the public health significance of this problem and the importance of IFA supplementation, no study has linked the level and factors associated with IFA supplementation compliance during pregnancy and its impact on post-pregnancy anaemia among women. So, in this study, we used propensity score matching (PSM) analysis on standard demographic and health survey (DHS) datasets from East African countries to highlight the importance of factors influencing IFA supplementation compliance, assess anaemia prevalence, and examine the impact of compliance on post-pregnancy anaemia among reproductive-age women.

## Methods

### Study design

The DHS programme employed a cross-sectional study design across selected countries in East Africa. The study adhered strictly to the Strengthening the Reporting of Observational Studies in Epidemiology (STROBE) guidelines (Supplemental Material).^49^

### Study setting and sampling

The DHS sampling was employed in two stages during the data collection period, which is hierarchal and cluster in nature. Firstly, enumeration areas or regions were selected and divided into urban and rural areas to create sampling strata. Enumeration areas were independently sampled within each stratum in two stages. Complete stratification and proportional allocation were done by sorting the sampling frame within each stratum and using probability proportional to size selection in the first stage. In the second stage, a fixed number of households per cluster was systematically selected. All women aged 15–49 who were either permanent residents or visitors who stayed the previous night were eligible for interviews.

This study analyses secondary data extracted from the recent standard DHS datasets of East African countries. According to the WHO, East Africa has 20 countries. Of these, only 10 countries (namely, Burundi, Ethiopia, Madagascar, Malawi, Mauritius, Rwanda, Tanzania, Uganda, Zambia and Zimbabwe) have recent standard DHS datasets for this analysis. Datasets from the following countries were excluded from this study due to the absence of recent standard DHS datasets (namely, Sudan, South Sudan, Djibouti, Comoros, Eritrea, Reunion, Seychelles, and Somalia) and required variables needed for this study (Mozambique and Kenya). The study included all standard DHS datasets collected from 2015 to 2022.

### Study population and eligibility criteria

Data were collected from all women aged 15–49 who were pregnant and had given birth before the survey period, focusing specifically on the index child/last-child.^50^ The study population included all women of the defined reproductive age from the selected enumeration areas in East African countries. A total of 43,200 participants were eligible for this study. Women who had given birth to at least one child before the survey period were eligible regardless of their pregnancy status at the time of the survey. However, if the data of the participants’ information lacked records of IFA supplementation or haemoglobin/anaemia, or with incomplete or missing survey responses were excluded from the study.

### Data sources

After a formal written request through https://dhsprogram.com/, the DHS programme allowed us to access the dataset of each East African country. The DHS programme collects data nationally across countries through a stratified multistage sampling technique. The survey collects information on population, health, and nutrition-related issues from individual to household levels using a detailed procedure which is published elsewhere.^50^ The DHS programme’s individual record (IR) dataset collects reproductive health data for the most recent pregnancy and delivery, specifically focusing on the last or index child from selected countries.

### Study variables

From each country’s IR dataset, we extracted the anaemia (outcome variable for PSM analysis), IFA supplementation compliance (the outcome variable for multilevel analysis and the exposure variable for PSM analysis), and independent variables (socio-demographic and reproductive health-related variables).

The socio-demographic variables were the mother’s age, marital status, husband’s educational status, husband’s occupational status, maternal occupational status, maternal educational status, head of the household, family size, residence (urban/rural), countries of participants and wealth status. Furthermore, other reproductive and other health-related variables were time of ANC visit, ANC utilization, history of teenage pregnancy, pregnancy status at the time of the survey, number of living children (parity), last pregnancy was wanted, ANC from a skilled provider (during last pregnancy), having health insurance coverage, mass media exposure, distance from health institution (providing ANC care) and history of hormonal contraceptive use.

Antenatal care services by skilled professionals for the most recent birth refer to care provided by doctors, nurses, midwives and auxiliary nurses/midwives.^50^ Post-pregnancy anaemia was defined using a cut-off value of <11.0 mg/dl for haemoglobin level, and in a non-pregnant woman, the cut-off value was <12.0 mg/dl.^51^ Post-pregnancy time is a period within 12 months after the birth of the last child. This study included datasets containing observations of both IFA supplementation and anaemia. In addition to this, anaemia status was determined and found within each country dataset after adjusting for altitude and smoking status.

IFA supplementation compliance was defined as the number of tablets consumed by pregnant women during pregnancy. A woman who took ⩾90 IFA supplementation tablets/capsules during her previous pregnancy was considered a complaint to IFA supplementation.^19,24–26,52^ The information regarding iron tablets or syrup intake includes any iron-containing supplementation, including IFA pills, multiple micronutrient tablets or powder and iron-only tablets.^18,50^

Furthermore, the wealth index is a cumulative measure of households’ living standards using household assets and income that was determined using principal component analysis. The wealth status within each country’s dataset was divided into five quantiles.^29^

### Statistical analysis

The data analysis and cleaning were employed using Stata version 17/MP (Stata Corporation IC., College Station, TX, USA) for Windows. Due to the hierarchical nature of the DHS data, we checked the presence of variation across clusters (presence of independence) using intra-class correlation coefficient (ICC) and deviance values. IFA supplementation compliance was determined as the number of women distributed by the number of days, they took IFA tablets or iron syrup during the pregnancy of their last birth (variable name: m45 and m46_1) (according to the guide to DHS statistics version 7.2).

Missing values in variables were managed as per the guide to the DHS statistics manual.^50^ We conducted a bi-variable multilevel mixed-effect binary logistic regression for the outcome variable (IFA supplementation compliance) that helps to identify statistically significant variables in predicting IFA supplementation compliance during multivariable analysis. Subsequently, multivariable multilevel mixed effect binary logistic regression was employed for variables with a *p*-value <0.25 during the bi-variable analysis to select candidate variables for subsequent PSM analysis.

Finally, we calculated adjusted odds ratios (AORs) with their corresponding 95% confidence intervals (CIs). By estimating the CIs of AORs, we determined the variables’ statistical significance and the strength of the association between IFA supplementation compliance and various independent variables.

### Parameter estimation method

In this study, ICC and median odd ratios (MORs) were used to determine the presence of variation between clusters and regions of each country. The ICC was calculated using the formula: ICC = $\frac{\left(\sigma \mu^{2}\right)}{\sigma \mu^{2}+\sigma e^{2}},$ where σμ^2^ is the variance of the group level; and σe^2^ denotes the variance of the individual level.^53^ Moreover, MOR was calculated as follows: MOR = exp $\sqrt{2\sigma^{2}*\Phi^{-1}(3/4)},$ where σ^2^ is the variance of the null model, and Φ^−1^ is the inverse of the standard normal cumulative distribution function.^54^

#### PSM analysis

The PSM analysis helped to assess the effect of IFA supplementation compliance on anaemia by considering the effect of other statistically significant variables among pregnant women. The PSM analysis was employed using StatNotesbook (a beta version 0.1.0) software for Windows. StatsNotebook is an open-source statistical package built on R. It is designed for reproducible analysis and interactive computing (https://statsnotebook.io/). Before doing the PSM analysis, we examined the statistical significance of the differences in proportions of anaemia between compliant and non-compliant groups with IFA supplementation using a chi-square test.

We performed the PSM analysis to ensure unbiased estimation between the compliant and non-compliant groups. The PSM technique has demonstrated the most substantial reduction in selection bias and residual biases when evaluating causal effects in observational studies,^20,55^ which helps to handle potential bias from non-random allocation of exposure in observational study designs.

After checking the association between IFA supplementation compliance during pregnancy and post-pregnancy anaemia (⩽12 months), we employed the PSM analysis using a one-to-one matching without replacement to determine the treatment effect of IFA supplementation compliance on anaemia among reproductive-age women.

The influence of IFA supplementation compliance during pregnancy on anaemia after pregnancy was estimated using a generalized linear model with a logit function (logistic regression) by using IFA supplementation compliance (yes/no) as a treatment variable, and statistically significant variables from model I^c^ as the explanatory variables.

## Result

### Socio-demographic characteristics of the participants

This study included data from 43,200 reproductive-age women (between 15 and 49 years of age). The mean ± SD (standard deviation) age of the study participants was 29.07 ± 7.23 years. Across all ranges, the highest proportions (68.25%) of the study participants were found between the ages of 20 and 34. Three-quarters of study participants resided in rural areas across East African countries. Among the study participants, 46% completed at least one grade in the primary education category (commonly, between grades 1 and 8) (Table 1).

**Table 1.** Socio-demographic characteristics of study participants in East African countries, 2015–2022 (*n* = 43,200).

Variables	Frequency	%
Mother’s age (in completed years)		
<20 years	3155	7.30
20–34 years	29,482	68.25
35–49 years	10,563	24.45
Marital status		
Not married	2766	6.40
Married	40,434	93.60
Husband’s educational status^a^		
No education	7780	22.28
Primary education	14,904	42.68
Secondary education and above	12,239	35.04
Husband occupational status^a^		
Not working	2987	8.54
Working	31,975	91.46
Maternal occupational status^a^		
Not working	15,431	36.08
Working	27,336	63.92
Maternal educational status		
No education	10,629	24.60
Primary education	19,877	46.01
Secondary education and above	12,694	29.39
Head of the household		
Male	32,042	74.17
Female	1158	25.83
Family size		
⩽6	28,500	65.97
>6	14,700	34.03
Residence		
Urban	10,881	25.19
Rural	32,319	74.81
Countries		
Burundi	4282	9.91
Ethiopia	6693	15.49
Madagascar	4736	10.96
Malawi	4354	10.08
Mauritania	2959	6.85
Rwanda	3093	7.16
Tanzania	2849	6.59
Uganda	3358	7.77
Zambia	6461	14.96
Zimbabwe	4415	10.22
Wealth index		
Poorer	19,182	44.40
Middle	8130	18.82
Richer	15,888	36.78

a Have a missing value (ranging from 0.68% to 19.13%).

### Reproductive health characteristics of the respondents

In this study, 36.44% of participants sought ANC at healthcare facilities before reaching 12 weeks of gestation during their pregnancy with their index child. Out of the total, 4990 women (11.55%) had health insurance. Additionally, 19,364 (44.82%) of the participants had a history of teenage pregnancy. Only a small proportion (8.48%) of the women had exposure to mass media like radio, newspapers and television.

Alarmingly, two out of five women faced difficulties in accessing healthcare facilities due to the distance between their homes and health facilities, which remains an ongoing challenge in developing countries. Two-fifths of the women considered the distance from home to the health institution to be a major problem. From all, 82.91% of the women received ANC services from skilled healthcare providers during their pregnancy period (Table 2).

**Table 2.** Reproductive and other health-related characteristics of study participants in East Africa, 2015–2022 (*n* = 43,200).

Variables	Frequency	%
Time of ANC visit (weeks)		
<12	15,744	36.44
⩾12	27,456	63.56
ANC utilization		
No	4054	9.38
Yes	38,929	90.11
Do not know	217	0.50
History of teenage pregnancy		
No	23,836	55.18
Yes	19,364	44.82
Currently pregnant		
No or unsure	39,203	90.75
Yes	3997	9.25
Number of living children (parity)		
⩽3	26,414	61.14
⩾4	16,786	38.86
Current pregnancy wanted		
Then	29,415	68.09
Later	10,343	23.94
Not at all	3442	7.97
ANC services from skilled healthcare providers		
No	7383	17.09
Yes	35,817	82.91
Have health insurance coverage		
No	38,210	88.45
Yes	4990	11.55
Mass media exposure		
No	39,538	91.52
Yes	3662	8.48
Distance from health institution		
Big problem	17,006	39.37
Not a big problem	26,194	60.63
History of hormonal contraceptive use		
No	31,082	71.95
Yes	12,118	28.05

ANC: antenatal care.

### Level of IFA supplementation compliance and prevalence of anaemia

The level (prevalence) of IFA supplementation compliance during pregnancy varied across the study countries in East Africa (Table 3). In East Africa, the overall level of IFA supplementation compliance during pregnancy was 31.33% (95% CI: 30.89, 31.77). The highest level of IFAS compliance was found in Zambia (79.14%), and the lowest was in Burundi (1.52%).

**Table 3.** Prevalence of anaemia and IFA supplementation compliance rate among RAW in each East African country, 2015–2022.

Countries	Survey year	Total number	Anaemia, frequency (%)	IFAS compliance^a^, frequency (%)
Burundi	2016/2017	4282	1793 (41.87)	65 (1.52)
Ethiopia	2016	6693	2193 (32.77)	456 (6.81)
Madagascar	2021	4736	1421 (30.00)	957 (20.21)
Mauritania	2019/2021	2959	1556 (52.59)	1066 (36.03)
Malawi	2015/2016	4354	1350 (31.01)	1597 (36.68)
Rwanda	2019/2020	3093	380 (12.29)	507 (16.39)
Tanzania	2022	2849	1167 (40.96)	1106 (38.82)
Uganda	2016	3358	1105 (32.91)	784 (23.35)
Zambia	2018/2019	6461	1806 (27.95)	5113 (79.14)
Zimbabwe	2015	4415	1086 (24.60)	1885 (42.70)
Total		43,200	13,857 (32.08)	13,536 (31.33)

IFA: iron–folic acid.

a A pregnant woman was considered to have IFAS compliance if she consumed 90 or more IFA supplements, tablets, or capsules throughout her pregnancy.

Additionally, the breakdowns of IFA supplement use during pregnancy were as follows: 24.24% (11,445) did not use IFA supplement, 35.90% (16,948) used it for <60 days, 10.47% (4942) used it for 60–89 days and 31.33% (13,871) used it for ⩾90 days. However, details in the forms of IFA supplementation, such as tablet, syrup, or any other types were not available in the dataset.

The mean ± SD haemoglobin level of the study participants was 12.45 ± 1.76 mg/dl. On the other hand, the average prevalence of anaemia among reproductive-age women was 32.08% (95% CI: 31.64, 32.52), which ranged from 12.29% (Rwanda) to 52.59% (Mauritania) as shown in Table 3.

#### Intra-class correlation assessment

Initially, the presence of correlation among the strata at regional and cluster levels/enumeration areas was determined using ICC and MOR results. The levels of ICC at regional and enumeration areas (cluster) were 43.52% (95% CI: 37.30, 49.95) and 52.36% (95% CI: 46.93, 57.73), respectively. The MOR results of the regional and cluster-level analysis indicate that compliance with IFA supplementation varied significantly across different regions and enumeration area clusters.

Pregnant women from regions (MOR = 5.82, 95% CI: 2.40, 14.10) and enumeration area clusters (MOR = 2.02, 95% CI: 1.88, 2.16), with a higher level of compliance with IFA supplementation, and had higher odds of compliance with IFA supplementation as compared to women from other regions or enumeration area clusters.

#### Factors associated with IFA supplementation compliance

Initially, bivariate multilevel mixed-effects logistic regression was used to identify factors associated with IFA supplementation compliance during pregnancy. The fourth model was the best fit, with the lowest deviance (−LL = 19288.96). The statistically significant factors from multivariable mixed-effects logistic regression were maternal education, time to start ANC follow-up, wanted the index pregnancy (last pregnancy), wealth status, distance from health institutions, mass media exposure and ANC service from skilled healthcare providers were found to be associated with IFA supplementation compliance during pregnancy among study participants in East Africa (see Table 4).

**Table 4.** Multivariable multilevel mixed-effects binary logistic regression analysis of factors associated with IFA supplementation compliance among reproductive-age women in East Africa, 2015–2021.

Variables	Model I,^a^ AOR (95% CI)	Model I,^b^ AOR (95% CI)	Model I,^c^ AOR (95% CI)
History of teenage pregnancy			
No	1		1
Yes	0.95 (0.90, 1.01)		0.96 (0.91, 1.03)
Age of the respondent (years)			
<20	1		1
20–34	0.93 (0.84, 1.04)		0.93 (0.83, 1.03)
35–49	0.98 (0.86, 1.13)		0.97 (0.85, 1.11)
Maternal educational status			
No formal education	1		1
Primary education	1.25 (1.15, 1.36)		1.21 (1.11, 1.32)*
Secondary education and above	1.66 (1.51, 1.84)		1.53 (1.38, 1.70)*
Parity			
<4	1		1
⩾4	0.93 (0.86, 1.00)		0.94 (0.88, 1.01)
Time to start ANC (weeks)			
<12	1		1
⩾12	0.57 (0.55, 0.61)		0.58 (0.55, 0.62)*
ANC services from a skilled healthcare provider			
No	1		1
Yes	3.01 (2.66, 3.41)		2.97 (2.61, 3.37)*
Pregnancy wanted (index child)			
Wanted	1		1
Later	0.83 (0.78, 0.89)		0.84 (0.79, 0.89)*
No more	0.80 (0.72, 0.89)		0.81 (0.72, 0.90)*
History of hormonal contraceptive use			
No	1		1
Yes	1.05 (0.99, 1.12)		1.05 (0.99, 1.11)
Insurance			
No		1	1
Yes		1.21 (1.07, 1.37)	1.07 (0.95, 1.22)
Distance from health institution			
Big problem		1	1
Not big problem		1.15 (1.08, 1.23)	1.11 (1.04, 1.19)*
Mass media exposure			
No		1	1
Yes		1.32 (1.21, 1.45)	1.18 (1.08, 1.30)*
Family size			
⩽6		1	1
>6		0.89 (0.84, 0.94)	0.97 (0.91, 1.03)
Wealth index			
Poorer		1	1
Middle		1.21 (1.11, 1.30)	1.12 (1.03, 1.21)*
Richer		1.35 (1.25, 1.47)	1.14 (1.04, 1.24)*
Residence			
Urban		1	1
Rural		1.02 (0.93, 1.13)	1.07 (0.98, 1.18)

NB: 1: Reference; CI: confidence interval; ANC: antenatal care; AOR: adjusted odds ratio; IFA: iron–folic acid.

a Adjusted for individual-level variables.

b Adjusted for household-level variables.

c Full model (adjusted for individual- and household-level variable.

* *p* < 0.001.

Maternal education significantly influenced compliance with IFA supplementation in pregnant women. A pregnant woman with primary education (AOR = 1.21, 95% CI: 1.11, 1.32) and secondary education or higher (AOR = 1.53, 95% CI: 1.38, 1.70) exhibited 1.21- and 1.53-times higher odds of being compliant, respectively.

Conversely, pregnant women initiating ANC visits after 12 weeks of pregnancy were 42% less likely to comply with IFA supplementation (AOR = 0.58, 95% CI: 0.55, 0.62) compared to those who started ANC earlier. Furthermore, pregnant women who wanted the pregnancy later (AOR = 0.84, 95% CI: 0.79, 0.89) or unwanted pregnancy at all (AOR = 0.81, 95% CI: 0.72, 0.90) were 16% and 19% less likely to have compliance with IFA supplementation, respectively, compared to those who were wanting the pregnancy.

Regarding wealth status, pregnant women from middle-income households were 1.12 times more likely to comply (AOR = 1.12, 95% CI: 1.03, 1.21), while those from rich households were 1.14 times more likely (AOR = 1.14, 95% CI: 1.04, 1.24), compared to pregnant women from a poor household.

Similarly, pregnant women who did not face significant distance barriers in accessing health institutions and those with mass media access had 1.11 times higher odds (AOR = 1.11, 95% CI: 1.08, 1.30) and 1.09 times higher odds (AOR = 1.09, 95% CI: 1.01, 1.19) of being compliant with IFA supplementation compared to their counterparts, respectively. Additionally, pregnant women receiving ANC services from skilled healthcare providers had 2.97 times higher odds of complying (AOR = 2.97, 95% CI: 2.61, 3.37) with IFA supplementation than those who did not.

#### Results of PSM analysis

For the PSM analysis, we used data from 13,675 reproductive-age women, 12 months postpartum, out of 43,200 to assess the effect of IFAS compliance during pregnancy on post-pregnancy anaemia prevalence. A chi-squared test revealed a significant association in post-pregnancy anaemia prevalence (*p* < 0.0001) based on IFA supplementation compliance.

Using statistically significant variables related to IFA supplementation compliance identified through mixed-effect logistic regression analysis from model I^c^ (as shown in Table 4), PSM analysis was employed to assess the influence of IFA supplementation compliance during pregnancy on anaemia after pregnancy among the women participants.

Then, we matched IFA supplementation compliance with non-compliant counterparts to assess its effect on anaemia after pregnancy. Each compliant individual receiving IFA supplementation (*n* = 4831) was paired/matched with a non-compliant counterpart at a one-to-one ratio (Supplemental Table 1 and Supplemental Figure 1). Subsequently, unmatched cases of non-compliance with IFA supplementation during pregnancy (*n* = 5061) were excluded from the PSM analysis.

Before matching, significant disparities existed in all covariates between compliant and non-compliant study participants with IFA supplementation. However, post-matching showed a well-balanced distribution of IFA supplementation between compliant and non-compliant pregnant women, with standardized mean differences approaching zero. This indicates a high degree of balance, as disparities were minimized after matching, aligning closely with or near the zero standardized mean difference line (Supplemental Figure 2).

The analysis indicated that compliance with IFA supplementation during pregnancy was associated with a 25.55% reduction in post-pregnancy anaemia compared with the non-compliant group (average treatment effect on the treated: −0.2555, 95% CI: −0.3440, −0.1669, *p* < 0.0001).

## Discussion

This study utilizes the standard DHS dataset from East African countries, collected between 2015 and 2022, to examine the factors influencing IFA supplementation compliance among women during their previous pregnancy and its impact on post-pregnancy anaemia. It reveals three key findings. Firstly, the overall level of compliance with IFA supplementation was quite low (31.33%), and one in three women was anaemic. Secondly, the level of compliance with IFA supplementation during pregnancy was statistically associated with maternal socio-demographic characteristics, reproductive health, and socio-economic-related variables. Third, the study demonstrated that complying with IFA supplementation during pregnancy lowers the prevalence of post-pregnancy anaemia by 25.55%.

In the present study, the level of compliance with IFA supplementation in East Africa was low and varied widely, with over two-thirds of pregnant women not complying, similar to the findings of studies from other developing and sub-Saharan African countries using DHS datasets.^30,56^ The low level of compliance with IFA supplementation may be due to insufficient stakeholder engagement,^57^ individual and community-level factors^58^ and temporary side effects of the supplement.^59^ Additionally, variations across regions and countries could be influenced by the DHS data being collected in different years.

In the present study, the odds of IFA supplementation compliance during pregnancy were higher among women with formal education than those without. This study finding is consistent with a similar study in Ethiopia^58^ and other sub-Saharan African countries^56^ using mini/interim and standard DHS datasets, respectively. Educated women may have a high level of awareness about pregnancy requirements, access to health education and readily follow medical advice/counselling from skilled healthcare workers.^60,61^ As a result, educated women may better understand the benefits of complying with IFA supplementation.

Our results showed that pregnant women starting ANC before 12 weeks of gestation for the last pregnancy were more likely to comply with IFA supplementation compared to their counterparts. This finding aligns with current WHO recommendations for the early initiation of ANC visits.^48^ Studies have also shown that early initiation of ANC visits increases the frequency of ANC visits and counselling sessions,^62–64^ which allows a longer duration to take the recommended IFA supplements (⩾90) during the pregnancy period.^30^ Furthermore, women who initiate ANC early are more likely to understand the benefits of IFA supplementation for both the foetus and pregnant women. This is due to frequent contact with skilled healthcare providers, giving them more time to be informed and encouraged by health professionals to take the IFA tablets.^48,58^

Furthermore, pregnant women who either wanted to delay their last pregnancy or did not want it at all were less likely to comply with IFA supplementation compared to those who wanted their pregnancy. Women’s desire for pregnancy may lead to planning for safe healthcare practices and a stronger commitment to maternal behaviour changes.^65–67^ Women who did not intend or desire their pregnancy may be less likely to access healthcare services, follow provider advice, attend regular ANC visits and engage in self-care practices related to pregnancy, including consumption of IFA supplements.^68–70^

We found that household wealth status was positively associated with IFA supplementation compliance during pregnancy among pregnant women. It is a fact that household socio-economic status impacts individual health, particularly among women in developing countries.^71,72^ This study result is consistent with study findings from Malawi,^64^ sub-Saharan African countries,^56^ Tanzania^16^ and Pakistan.^73^ A likely explanation is that wealth status influences access to healthcare services and the ability to purchase IFA tablets, especially when these tablets are not available at health facilities during ANC visits as shown in the study in Hawassa City Ethiopia^59^ and across different developing countries.^30^ Additionally, healthcare coverage and service inequality vary across sub-Saharan and East African countries,^74–76^ with less than 50% of women in East Africa accessing healthcare institutions between 2008 and 2017.^77^

Our finding also revealed that when the distance from home to health institutions was not a big problem, women were more compliant with IFA supplementation during pregnancy. Studies from Bangladesh,^78^ South Africa,^79^ Ghana,^80^ Holeta Town Central Ethiopia^81^ and Kenya,^82^ showed that distance from home to health institutions affects access to maternal healthcare services like ANC follow-ups. The likely reason is that walking long distances is difficult during pregnancy, and adequate transportation to health institutions is also a challenge in most developing countries as revealed by a systematic review.^83^ Moreover, pregnant women may acquire knowledge through peer observation, particularly when observing peers seek ANC services at nearby health institutions if the accessibility issues related to distance are not a barrier.^84^

Furthermore, our study found that pregnant women who had access to mass media (TV, newspaper, radio, etc.) were more likely to have IFA supplementation compliance. Also, pregnant women who obtained ANC services from skilled healthcare providers were more likely to comply with IFA supplementation, which is like the findings of previous studies.^85,86^ In low-resource settings, mass media and trained healthcare workers play a role as the sources of information,^62,63^ which might increase maternal satisfaction and knowledge about the benefits of IFA supplementation during pregnancy. Therefore, these pregnant women are more likely to take the recommended dose of IFA supplementation. Moreover, women with better healthcare access tend to receive more information about the benefits of IFA supplementation^58,87^ through health campaigns and frequent ANC visits.

Finally, we examined the effect of IFA supplementation compliance during pregnancy on the prevalence of post-pregnancy anaemia, ensuring a balance of factors between the compliant and non-compliant groups to obtain unbiased estimates from survey data. The PSM analysis in this study indicated that women who were compliant with IFA supplementation had a lower risk of anaemia during post-pregnancy, which aligns with the recommendations in WHO micronutrient supplementation guidelines ^18,19^; however, we could not identify a similar study to facilitate further discussion. Compliance with IFA supplementation during pregnancy might enhance reserves or stores of iron that can prevent iron deficiency or iron-deficiency anaemia in later life.^60^

### Study strengths and limitations

The major strength of this study is the use of nationally representative data/samples from 10 East African countries. This is one of the few studies^16,56,58,88^ using DHS data to assess IFA supplementation compliance and its effect on anaemia after pregnancy among women in East Africa. PSM analysis facilitates comparing compliant and non-compliant groups with similar socio-demographic characteristics and possesses similar potential influencing factors without bias. This method offers an unbiased estimate of the relationship between IFA compliance and anaemia occurrence by reducing confounding variables and biases compared to traditional logistic regression models.

Our study has certain limitations. It relies on cross-sectional survey datasets, limiting our ability to establish definitive cause-and-effect relationships between factors and IFA supplementation compliance. Additionally, some East African countries were excluded from our study due to the lack of data for the required dependent variables (IFA supplementation compliance and anaemia) or outdated datasets. Additionally, we did not perform a sample size calculation or power analysis, as we utilized secondary data that met the eligibility criteria.

We acknowledge the limitations in addressing all important variables related to participant (individual), contextual and infrastructural variables. So, we highly recommend conducting research that minimizes these limitations and addresses the distribution and accessibility of IFA supplementation.

### Implications for practice

Stakeholders’ efforts to improve IFA supplementation compliance during pregnancy are essential for addressing multiple interrelated gaps with strategies. Enhanced compliance helps prevent postpartum anaemia by replenishing maternal iron levels depleted during pregnancy and childbirth. It also builds sufficient iron stores in women of reproductive age, preparing them for future pregnancies and reducing the risk of maternal complications. Furthermore, adequate iron intake during pregnancy supports foetal development and reduces the likelihood of anaemia in newborns, which can affect their growth and cognitive development. These combined effects underscore the importance of targeted interventions, education and healthcare support to ensure pregnant women adhere to IFA supplementation guidelines. Regular monitoring and evaluation are also essential to identify and address implementation gaps.

### Future research

Comprehensive research is needed to explore gaps in service implementation, nutrition service quality, healthcare providers’ knowledge and the behaviours of pregnant women to improve IFA supplementation compliance during pregnancy to lower the risk of anaemia during post-pregnancy.

## Conclusion

The overall prevalence of anaemia among the study participants was high. Also, the level of compliance with IFA supplementation was found low in East African countries. Here, both individual and socio-economic status-related variables were associated with the IFA supplementation compliance among pregnant women.

A significant positive association was observed between anaemia status and IFA supplementation compliance during pregnancy, highlighting the importance of effective IFA implementation.

## Supplemental Material

sj-docx-1-whe-10.1177_17455057251317547 – Supplemental material for Iron and folic acid supplementation compliance during pregnancy and its effect on post-pregnancy anaemia among reproductive-age women in East Africa Supplemental material, sj-docx-1-whe-10.1177_17455057251317547 for Iron and folic acid supplementation compliance during pregnancy and its effect on post-pregnancy anaemia among reproductive-age women in East Africa by Melaku Tadege Engidaw, Patricia Lee and Faruk Ahmed in Women’s Health

sj-docx-2-whe-10.1177_17455057251317547 – Supplemental material for Iron and folic acid supplementation compliance during pregnancy and its effect on post-pregnancy anaemia among reproductive-age women in East Africa Supplemental material, sj-docx-2-whe-10.1177_17455057251317547 for Iron and folic acid supplementation compliance during pregnancy and its effect on post-pregnancy anaemia among reproductive-age women in East Africa by Melaku Tadege Engidaw, Patricia Lee and Faruk Ahmed in Women’s Health

sj-docx-3-whe-10.1177_17455057251317547 – Supplemental material for Iron and folic acid supplementation compliance during pregnancy and its effect on post-pregnancy anaemia among reproductive-age women in East Africa Supplemental material, sj-docx-3-whe-10.1177_17455057251317547 for Iron and folic acid supplementation compliance during pregnancy and its effect on post-pregnancy anaemia among reproductive-age women in East Africa by Melaku Tadege Engidaw, Patricia Lee and Faruk Ahmed in Women’s Health

sj-docx-4-whe-10.1177_17455057251317547 – Supplemental material for Iron and folic acid supplementation compliance during pregnancy and its effect on post-pregnancy anaemia among reproductive-age women in East Africa Supplemental material, sj-docx-4-whe-10.1177_17455057251317547 for Iron and folic acid supplementation compliance during pregnancy and its effect on post-pregnancy anaemia among reproductive-age women in East Africa by Melaku Tadege Engidaw, Patricia Lee and Faruk Ahmed in Women’s Health

## Declarations

**Ethics approval and consent to participate:** We have obtained authorization to analyse each country dataset from the DHS programme, ensuring that personal identifiers are absent. Given that this data is secondary and openly available, the requirement for consent to participate and for publication is waived. The datasets have been managed in compliance with the Helsinki Declaration by accessing anonymized data, securing permission from the DHS programme to utilize the datasets and adhering to ethical protocols throughout the process.

**Consent for publication:** We received approval from DHS to publish the de-identified data, and obtaining consent from the study participants is not applicable since we used de-identified secondary data.

**Availability of data and materials:** The datasets are accessible to the public with permission for download from The DHS Programme. Anyone can obtain each country dataset through https://dhsprogram.com/. The compiled dataset used and/or analysed for this study is available from the corresponding author upon reasonable request and permission from DHS data archivists.

## References

[1] WHO. WHO global anaemia estimates, 2021 edition, 2021. https://www.who.int/data/gho/data/themes/topics/anaemia_in_women_and_children.

[2] De Benoist B Cogswell M Egli I , et al. Worldwide prevalence of anaemia 1993–2005; WHO Global Database of anaemia. Geneva: World Health Organization, 2008;

[3] Osungbade KO Oladunjoye AO. Anaemia in developing countries: burden and prospects of prevention and control. Anemia 2012; 3: 116–129.

[4] Stevens GA Finucane MM De-Regil LM , et al. Global, regional, and national trends in haemoglobin concentration and prevalence of total and severe anaemia in children and pregnant and non-pregnant women for 1995–2011: a systematic analysis of population-representative data. Lancet Glob Health 2013; 1(1): e16–e25. 10.1016/S2214-109X(13)70001-9 PMC4547326 25103581

[bibr5-17455057251317547] Shekar M Condo J Pate MA , et al. Maternal and child undernutrition: progress hinges on supporting women and more implementation research. Lancet 2021; 397(10282): 1329–1331. 33691093 10.1016/S0140-6736(21)00577-8

[6] Kakietek J Eberwein JD Walters D , et al. Unleashing gains in economic productivity with investments in nutrition. Washington, DC: World Bank Group, 2017.

[7] Hatzopoulou K Filis V Grammatikopoulou MG , et al. Greek pregnant women demonstrate inadequate micronutrient intake despite supplement use. J Diet Suppl 2014; 11(2): 155–165. 24670119 10.3109/19390211.2013.859210

[8] Beard J. Iron deficiency alters brain development and functioning. J Nutr 2003; 133(5): 1468S–1472S. 10.1093/jn/133.5.1468S 12730445

[bibr9-17455057251317547] Braunstein GD . MSD Manual Professional Edition. Etiology of Anemia – Hematology and Oncology, May 2024. https://www.msdmanuals.com/professional/hematology-and-oncology/approach-to-the-patient-with-anemia/etiology-of-anemia.

[10] Chaparro CM Suchdev PS. Anemia epidemiology, pathophysiology, and etiology in low- and middle-income countries. Ann N Y Acad Sci 2019; 1450(1): 15–31. 31008520 10.1111/nyas.14092 PMC6697587

[11] Alexander L. Tackling iron deficiency in developing countries. The Borgen Project, https://borgenproject.org/tackling-iron-deficiency-in-developing-countries/ (2020).

[12] Hasan MM Soares Magalhaes RJ Garnett SP , et al. Anaemia in women of reproductive age in low- and middle-income countries: progress towards the 2025 global nutrition target. Bull World Health Organ 2022; 100(3): 196–204. 35261408 10.2471/BLT.20.280180 PMC8886255

[bibr13-17455057251317547] Kalaivani K. Prevalence & consequences of anaemia in pregnancy. Indian J Med Res 2009; 130(5): 627–633. 20090119

[bibr14-17455057251317547] Steer PJ. Maternal hemoglobin concentration and birth weight. Am J Clin Nutr 2000; 71(5): 1285S–1287S. 10799403 10.1093/ajcn/71.5.1285s

[15] Zhang Q Ananth CV Rhoads GG , et al. The impact of maternal anemia on perinatal mortality: a population-based, prospective cohort study in China. Ann Epidemiol 2009; 19(11): 793–799. 19648029 10.1016/j.annepidem.2009.06.002

[16] Ogundipe O Hoyo C Stbye T , et al. Factors associated with prenatal folic acid and iron supplementation among 21,889 pregnant women in Northern Tanzania: a cross-sectional hospital-based study. BMC Public Health 2012; 12: 481. 22734580 10.1186/1471-2458-12-481 PMC3438116

[17] Serrano NC Quintero-Lesmes DC Becerra-Bayona S , et al. Association of pre-eclampsia risk with maternal levels of folate, homocysteine and vitamin B12 in Colombia: a case-control study. PLoS One 2018; 13(12): e0208137. 10.1371/journal.pone.0208137 PMC6283543 30521542

[18] WHO. Antenatal iron supplementation, https://www.who.int/data/nutrition/nlis/info/antenatal-iron-supplementation (accessed 8 August 2023).

[bibr19-17455057251317547] World Health Organization. Guideline: daily iron and folic acid supplementation in pregnant women. Geneva: World Health Organization, 2012. 23586119

[bibr20-17455057251317547] Williamson E Morley R Lucas A , et al. Propensity scores: from naïve enthusiasm to intuitive understanding. Stat Methods Med Res 2012; 21(3): 273–293. 21262780 10.1177/0962280210394483

[21] World Health Organization. Guideline: daily iron supplementation in adult women and adolescent girls. Geneva: World Health Organization, 2016. 27195351

[22] United Nations Department of Economic and Social Affairs. Sustainable Development Goal 2: End hunger, achieve food security and improved nutrition and promote sustainable agriculture, https://sdgs.un.org/goals/goal2#targets_and_indicators (accessed 8 August 2023). 10.4102/jamba.v9i1.350 PMC6014178 29955332

[23] Ansu-Mensah M Danquah FI Bawontuo V , et al. Maternal perceptions of the quality of Care in the Free Maternal Care Policy in sub-Sahara Africa: a systematic scoping review. BMC Health Serv Res 2020; 20(1): 911. 33004029 10.1186/s12913-020-05755-9 PMC7528345

[24] World Health Organization. Developing and validating an iron and folic acid supplementation indicator for tracking progress towards global nutrition monitoring framework targets: final report June 2018. Geneva: World Health Organization, 2018.

[bibr25-17455057251317547] World Health Organization. Global Nutrition Monitoring Framework: operational guidance for tracking progress in meeting targets for 2025. Geneva: World Health Organization, 2017.

[26] Nutrition International. Iron-Folic Acid Supplementation in Pregnancy: Program Logic Model and Key Considerations for Program Success, 2019. https://www.nutritionintl.org/wp-content/uploads/2019/11/NI-IFA-Program-logic-model-and-considerations.pdf (accessed 8 August 2023).

[27] Abraha I Bonacini MI Montedori A , et al. Oral iron-based interventions for prevention of critical outcomes in pregnancy and postnatal care: an overview and update of systematic reviews. J Evid Based Med 2019; 12(2): 155–166. 31144465 10.1111/jebm.12344

[28] Peña-Rosas JP De-Regil LM Garcia-Casal MN , et al. Daily oral iron supplementation during pregnancy. Cochrane Database Syst Rev 2015; 2015(7): CD004736. 10.1002/14651858.CD004736.pub5 PMC8918165 26198451

[29] Lassi ZS Kedzior SG Tariq W , et al. Effects of preconception care and periconception interventions on maternal nutritional status and birth outcomes in low-and middle-income countries: a systematic review. Nutrients 2020; 12(3): 606. 32110886 10.3390/nu12030606 PMC7146400

[30] Sununtnasuk C D’Agostino A Fiedler JL. Iron+folic acid distribution and consumption through antenatal care: identifying barriers across countries. Public Health Nutr 2016; 19(4): 732–742. 26022914 10.1017/S1368980015001652 PMC10270815

[31] Woldegebriel AG Gebregziabiher Gebrehiwot G Aregay Desta A , et al. Determinants of anemia in pregnancy: findings from the Ethiopian Health and Demographic Survey. Anemia 2020; 2020: 2902498. 32566286 10.1155/2020/2902498 PMC7293722

[32] CSA Ethiopia and ICF International: Mini Ethiopia Demographic and Health Survey 2019. Addis Ababa Ethiopia and Calverton, Maryland, USA: Central statistical Agency and ICF International, 2019.

[bibr33-17455057251317547] Gulema H Berhane Y. Timing of first antenatal care visit and its associated factors among pregnant women attending public health facilities in Addis Ababa, Ethiopia. Ethiop J Health Sci 2017; 27(2): 139–146. 28579709 10.4314/ejhs.v27i2.6 PMC5440828

[34] Ministry of Health. Accelerating reduction of iron deficiency anaemia among pregnant women in Kenya: plan of action 2012–2017. Kenya: Division of Nutrition, 2012.

[35] UNICF. UNICEF Programming Guidance; Prevention of malnutrition in women before and during pregnancy and while breastfeeding. New York: UNICEF, 2021.

[36] Kavle JA Landry M. Addressing barriers to maternal nutrition in low- and middle-income countries: a review of the evidence and programme implications. Matern Child Nutr 2018; 14(1): e12508. 10.1111/mcn.12508 PMC5763330 28836343

[37] Kassa ZY Awraris T Daba AK , et al. Compliance with iron folic acid and associated factors among pregnant women through pill count in Hawassa city, South Ethiopia: a community based cross-sectional study. Reprod Health 2019; 16(1): 14. 30736812 10.1186/s12978-019-0679-8 PMC6367743

[bibr38-17455057251317547] Khorshid MR Afshari P Abedi P. The effect of SMS messaging on the compliance with iron supplementation among pregnant women in Iran: a randomized controlled trial. J Telemed Telecare 2014; 20(4): 201–206. 24803276 10.1177/1357633X14533895

[bibr39-17455057251317547] Saxena V Naithani M Kumari R , et al. Peri-conceptional supplementation of folic acid-knowledge and practices of pregnant women and health providers. J Fam Med Prim Care 2016; 5(2): 387. 10.4103/2249-4863.192374 PMC5084567 27843847

[bibr40-17455057251317547] Siabani S Siabani S Siabani H , et al. Determinants of compliance with iron and folate supplementation among pregnant women in West Iran: a population based cross-sectional study. J Fam Reprod Health 2018; 12(4): 197. PMC6581656 31239847

[bibr41-17455057251317547] Ugwu EO Olibe AO Obi SN , et al. Determinants of compliance to iron supplementation among pregnant women in Enugu, Southeastern Nigeria. Niger J Clin Pract 2014; 17(5): 608–612. 25244272 10.4103/1119-3077.141427

[42] Siekmans K Roche M Kung’u JK , et al. Barriers and enablers for iron folic acid (IFA) supplementation in pregnant women. Matern Child Nutr 2018; 14(Suppl 5): e12532. 10.1111/mcn.12532 PMC6865983 29271115

[43] Milman N. Postpartum anemia I: definition, prevalence, causes, and consequences. Ann Hematol 2011; 90(11): 1247–1253. 21710167 10.1007/s00277-011-1279-z

[44] WHO. Guideline: iron supplementation in postpartum women. Geneva: World Health Organization, 2016. 27583315

[bibr45-17455057251317547] Milman N. Anemia – still a major health problem in many parts of the world! Ann Hematol 2011; 90(4): 369–377. 21221586 10.1007/s00277-010-1144-5

[46] Moya E Phiri N Choko AT , et al. Effect of postpartum anaemia on maternal health-related quality of life: a systematic review and meta-analysis. BMC Public Health 2022; 22(1): 364. 35189871 10.1186/s12889-022-12710-2 PMC8862508

[47] Yefet E Yossef A Nachum Z. Prediction of anemia at delivery. Sci Rep 2021; 11(1): 6309. 33737646 10.1038/s41598-021-85622-7 PMC7973554

[48] World Health Organization. WHO recommendations on antenatal care for a positive pregnancy experience. Geneva: World Health Organization, 2016. 28079998

[49] Cuschieri S. The STROBE guidelines. Saudi J Anaesth 2019; 13(Suppl 1): S31. 10.4103/sja.SJA_543_18 PMC6398292 30930717

[50] Croft TN Marshall AM Allen CK , et al. Guide to DHS statistics. Rockville, MD, USA: ICF, 2018.

[51] World Health Organization. Haemoglobin concentrations for the diagnosis of anaemia and assessment of severity. Vitamin and mineral nutrition information system. Document reference WHO. NMH/NHD/MNM/11.1, http://www.who.int/entity/vmnis/indicators/haemoglobin (2011).

[52] The Nemours Foundation. Folic acid and pregnancy (for parents) – nemours KidsHealth, https://kidshealth.org/en/parents/preg-folic-acid.html (accessed 18 July 2023).

[53] Hesketh SR Skrondal A . Multilevel and longitudinal modeling using Stata. USA: STATA Press, 2021.

[54] Goldstein H . Multilevel statistical models, vol. 922. UK: John Wiley & Sons, 2011.

[55] Williamson EJ Forbes A. Introduction to propensity scores. Respirology 2014; 19(5): 625–635. 24889820 10.1111/resp.12312

[56] Ba DM Ssentongo P Kjerulff KH , et al. Adherence to iron supplementation in 22 Sub-Saharan African countries and associated factors among pregnant women: a large population-based study. Curr Dev Nutr 2019; 3(12): nzz120. 10.1093/cdn/nzz120 PMC6867960 31777771

[57] Sanghvi TG Nguyen PH Forissier T , et al. Comprehensive approach for improving adherence to prenatal iron and folic acid supplements based on intervention studies in Bangladesh, Burkina Faso, Ethiopia, and India. Food Nutr Bull 2023; 44(3): 183–194. 37309106 10.1177/03795721231179570

[58] Yalew M Getachew S Mohammed K , et al. Individual and contextual-level factors associated with iron-folic acid supplement intake during pregnancy in Ethiopia: a multi-level analysis. BMC Pregnancy Childbirth 2023; 23(1): 260. 37072714 10.1186/s12884-023-05593-7 PMC10111777

[59] Merse F Fanta A Kelbiso L. IFA utilization and factors associated with IFA utilization among pregnant women attending ANC at government health facilities and family guidance clinic in Hawassa city, South Ethiopia, https://www.stephypublishers.com/sojmccr/pdf/SOJMCCR.MS.ID.000512.pdf (2022).

[60] Smitha Mv Indumathi P Parichha S , et al. Compliance with IFA supplementation and its barriers in postpartum women in Odisha, India. Rochester, NY, https://papers.ssrn.com/abstract=4626996 (2023).

[61] Khanam A Vohra K Mg TA , et al. A systematic review of factors affecting compliance toward oral iron-folic acid supplementation among pregnant women in India. Indian J Community Health 2022; 34(4): 456–463.

[62] Haile MT Jeba AB Hussen MA. Compliance to prenatal iron and folic acid supplement and associated factors among women during pregnancy in south east Ethiopia: a cross-sectional study. J Nutr Health Food Eng 2017; 7(2): 272–277.

[bibr63-17455057251317547] Gebremedhin S Samuel A Mamo G , et al. Coverage, compliance and factors associated with utilization of iron supplementation during pregnancy in eight rural districts of Ethiopia: a cross-sectional study. BMC Public Health 2014; 14: 607. 24930036 10.1186/1471-2458-14-607 PMC4073172

[64] Titilayo A Palamuleni ME Omisakin O. Sociodemographic factors influencing adherence to antenatal iron supplementation recommendations among pregnant women in Malawi: analysis of data from the 2010 Malawi Demographic and Health Survey. Malawi Med J 2016; 28(1): 1–5. 27217909 10.4314/mmj.v28i1.1 PMC4864384

[65] Kost K Lindberg L. Pregnancy intentions, maternal behaviors, and infant health: investigating relationships with new measures and propensity score analysis. Demography 2015; 52(1): 83–111. 25573169 10.1007/s13524-014-0359-9 PMC4734627

[bibr66-17455057251317547] Gipson JD Koenig MA Hindin MJ. The effects of unintended pregnancy on infant, child, and parental health: a review of the literature. Stud Fam Plann 2008; 39(1): 18–38. 18540521 10.1111/j.1728-4465.2008.00148.x

[67] Kost K Landry DJ Darroch JE. The effects of pregnancy planning status on birth outcomes and infant care. Fam Plann Perspect 1998; 30(5): 223–230. 9782045

[68] Widowati LP Damayanti R. Unintended pregnancy and antenatal care behavior in Indonesia. JNKI J Ners Dan Kebidanan Indones Indones J Nurs Midwifery 2022; 10(3): 214–223.

[bibr69-17455057251317547] Carlander A Hultstrand JN Reuterwall I , et al. Unplanned pregnancy and the association with maternal health and pregnancy outcomes: a Swedish cohort study. PLoS One 2023; 18(5): e0286052. 10.1371/journal.pone.0286052 PMC10202275 37216351

[70] Khan MN Harris ML Shifti DM , et al. Effects of unintended pregnancy on maternal healthcare services utilization in low- and lower-middle-income countries: systematic review and meta-analysis. Int J Public Health 2019; 64(5): 743–754. 31041453 10.1007/s00038-019-01238-9

[71] Gwatkin DR Rutstein S Johnson K , et al. Socio-economic differences in health, nutrition, and population within developing countries: an overview. Niger J Clin Pract 2007; 10(4): 272–282. 18293634

[72] Houweling TA Kunst AE Mackenbach JP. Measuring health inequality among children in developing countries: does the choice of the indicator of economic status matter? Int J Equity Health 2003; 2(1): 1–12. 14609435 10.1186/1475-9276-2-8 PMC272937

[73] Nisar YB Dibley MJ Mir AM. Factors associated with non-use of antenatal iron and folic acid supplements among Pakistani women: a cross sectional household survey. BMC Pregnancy Childbirth 2014; 14: 305. 25189220 10.1186/1471-2393-14-305 PMC4162926

[74] Ogundipe OM Olurinola OI Ogundipe A. Health interventions and child health in Sub-Saharan Africa: assessing the impact of the millennium development goal. J Sustain Dev 2016; 9(1): 187.

[bibr75-17455057251317547] Bobo FT Asante A Woldie M , et al. Poor coverage and quality for poor women: Inequalities in quality antenatal care in nine East African countries. Health Policy Plan 2021; 36(5): 662–672. 33822943 10.1093/heapol/czaa192

[76] Medic East Africa. Universal Health Coverage in East Africa. 2019, p. 11, https://www.mediceastafrica.com/content/dam/Informa/mediceastafrica/2019/downloads/universal-health-coverage-east-africa.pdf (accessed 31 July 2024) .

[77] Minyihun A Tessema ZT. Determinants of access to health care among women in East African countries: a multilevel analysis of recent demographic and health surveys from 2008 to 2017. Risk Manag Healthc Policy 2020; 13: 1803–1813. 33061713 10.2147/RMHP.S263132 PMC7533273

[78] Keya KT Rob U Rahman MM , et al. Distance, transportation cost, and mode of transport in the utilization of facility-based maternity services: evidence from rural Bangladesh. Int Q Community Health Educ 2014; 35(1): 37–51. 25416431 10.2190/IQ.35.1.d

[79] McCray TM. An issue of culture: the effects of daily activities on prenatal care utilization patterns in rural South Africa. Soc Sci Med 2004; 59(9): 1843–1855. 15312919 10.1016/j.socscimed.2004.02.033

[80] Dotse-Gborgbortsi W Dwomoh D Alegana V , et al. The influence of distance and quality on utilisation of birthing services at health facilities in Eastern Region, Ghana. BMJ Glob Health 2020; 4(Suppl 5): e002020. 10.1136/bmjgh-2019-002020 PMC7044703 32154031

[81] Birmeta K Dibaba Y Woldeyohannes D. Determinants of maternal health care utilization in Holeta town, central Ethiopia. BMC Health Serv Res 2013; 13(1): 256. 23822155 10.1186/1472-6963-13-256 PMC3710264

[82] Asweto CO Aluoch JR Obonyo CO , et al. Maternal autonomy, distance to health care facility and ANC attendance findings from Madiany Division of Siaya County, Kenya. Am J Public Health Res 2014; 2(4): 153–158.

[83] Yunitasari E Putri DUW Armini NKA , et al. Analysis of factors that affect the utilization of antenatal care in developing countries: a systematic review. J Pak Med Assoc 2023; 73(2): S162–S169. 10.47391/JPMA.Ind-S2-37 37096726

[84] Putri AP Hanifah L Am AI , et al. Maternal health literacy among pregnant women in Indonesia: a qualitative study. Int J Nurs Health Serv 2023; 6(6): 354–364.

[85] Warvadekar K Reddy JC Sharma S , et al. Socio-demographic and economic determinants of adherence to iron intake among pregnant women in selected low and lower middle income countries in Asia: insights from a cross-country analyses of global demographic and health surveys. Int J Community Med Public Health 2018; 5(4): 1552–1569.

[86] Chourasia A Pandey CM Awasthi A. Factors influencing the consumption of iron and folic acid supplementations in high focus states of India. Clin Epidemiol Glob Health 2017; 5(4): 180–184.

[87] Say L Raine R. A systematic review of inequalities in the use of maternal health care in developing countries: examining the scale of the problem and the importance of context. Bull World Health Organ 2007; 85(10): 812–819. 18038064 10.2471/BLT.06.035659 PMC2636485

[88] Karyadi E Reddy JC Dearden KA , et al. Antenatal care is associated with adherence to iron supplementation among pregnant women in selected low-middle-income-countries of Asia, Africa, and Latin America & the Caribbean regions: insights from Demographic and Health Surveys. Matern Child Nutr 2023; 19(2): e13477. 10.1111/mcn.13477 PMC10019046 36705031

