# Supplementary material for: Iron and folic acid supplementation compliance during pregnancy and its effect on post-pregnancy anaemia among reproductive-age women in East Africa
Source: Womens Health (Lond). 2025 Feb 10;21:17455057251317547. doi: 10.1177/17455057251317547 (PMC11811972; doi:10.1177/17455057251317547)
Supplement: sj-docx-1-whe-10.1177_17455057251317547 – Supplemental material for Iron and folic acid supplementation compliance during pregnancy and its effect on post-pregnancy anaemia among reproductive-age women in East Africa [file sj-docx-1-whe-10.1177_17455057251317547.docx]

**Supplementary Table 1: Summary table for propensity matching score analysis of reproductive-age women in East Africa, 2015 – 2022.**

| **Sample size** | **of reproductive-age women (n= 13675)** | | **Total** |
| --- | --- | --- | --- |
|  | **Control** | **Treated** |  |
| All sample size | 9368 | 4307 | 13675 |
| Matched sample size | 4307 | 4307 | 8614 |
| Unmatched sample size | 5061 | 0 | 5061 |
| Discarded | 0 | 0 | 0 |
