# Supplementary material for: Iron and folic acid supplementation compliance during pregnancy and its effect on post-pregnancy anaemia among reproductive-age women in East Africa
Source: Womens Health (Lond). 2025 Feb 10;21:17455057251317547. doi: 10.1177/17455057251317547 (PMC11811972; doi:10.1177/17455057251317547)
Supplement: sj-docx-3-whe-10.1177_17455057251317547 – Supplemental material for Iron and folic acid supplementation compliance during pregnancy and its effect on post-pregnancy anaemia among reproductive-age women in East Africa [file sj-docx-3-whe-10.1177_17455057251317547.docx]

**Supplementary Figure 2: Standardized mean difference analysis**

**
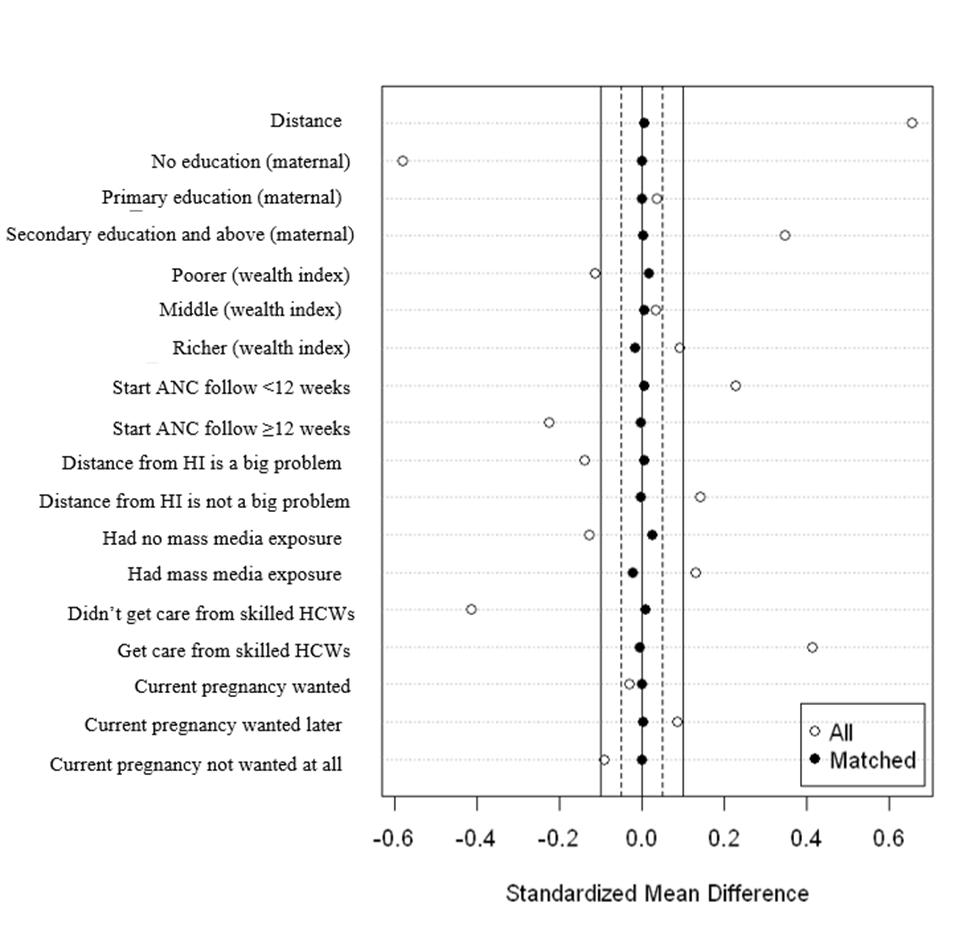
**

**Figure 2**: Standardized mean difference before and after PSM comparing covariates values for reproductive-age women who were compliant and non-compliant to IFA supplementation in East Africa, 2015 – 2022.
